# Functional enhancement strategies to potentiate the therapeutic properties of mesenchymal stromal cells for respiratory diseases

**DOI:** 10.3389/fphar.2023.1067422

**Published:** 2023-03-16

**Authors:** Miquéias Lopes-Pacheco, Patricia R. M. Rocco

**Affiliations:** ^1^ Biosystems & Integrative Sciences Institute, Faculty of Sciences, University of Lisbon, Lisbon, Portugal; ^2^ Laboratory of Pulmonary Investigation, Carlos Chagas Filho Institute of Biophysics, Federal University of Rio de Janeiro, Rio de Janeiro, Brazil

**Keywords:** cell therapy, genetic engineering, immunomodulation, inflammation, lung, preconditioning, remodeling

## Abstract

Respiratory diseases remain a major health concern worldwide because they subject patients to considerable financial and psychosocial burdens and result in a high rate of morbidity and mortality. Although significant progress has been made in understanding the underlying pathologic mechanisms of severe respiratory diseases, most therapies are supportive, aiming to mitigate symptoms and slow down their progressive course but cannot improve lung function or reverse tissue remodeling. Mesenchymal stromal cells (MSCs) are at the forefront of the regenerative medicine field due to their unique biomedical potential in promoting immunomodulation, anti-inflammatory, anti-apoptotic and antimicrobial activities, and tissue repair in various experimental models. However, despite several years of preclinical research on MSCs, therapeutic outcomes have fallen far short in early-stage clinical trials for respiratory diseases. This limited efficacy has been associated with several factors, such as reduced MSC homing, survival, and infusion in the late course of lung disease. Accordingly, genetic engineering and preconditioning methods have emerged as functional enhancement strategies to potentiate the therapeutic actions of MSCs and thus achieve better clinical outcomes. This narrative review describes various strategies that have been investigated in the experimental setting to functionally potentiate the therapeutic properties of MSCs for respiratory diseases. These include changes in culture conditions, exposure of MSCs to inflammatory environments, pharmacological agents or other substances, and genetic manipulation for enhanced and sustained expression of genes of interest. Future directions and challenges in efficiently translating MSC research into clinical practice are discussed.

## 1 Introduction

Respiratory diseases are primarily caused by genetic and environmental factors and/or social behaviors that lead to lung inflammation, injury, and remodeling, resulting in progressive deterioration of lung function and, ultimately, in respiratory failure (Aghasafari et al., 2019). These diseases are major health concerns worldwide because they result in a high rate of morbidity and disability, and subject patients to substantial financial and psychosocial burdens (Rehman et al., 2020; Soriano et al., 2020). Moreover, chronic obstructive pulmonary disease (COPD) and lower respiratory infections are two of the five leading causes of death worldwide, claiming over 6 million lives in 2019 according to data from the World Health Organization (World Health Organization, 2020). Despite remarkable progress in better understanding the pathologic mechanisms underlying severe respiratory diseases, the currently available therapies aim to alleviate symptoms and/or delay their natural progressive course but are unable to reverse morphologic and functional abnormalities already established in the lungs.

Mesenchymal stromal cells (MSCs) have been considered a versatile source for cell-based therapies and are at the forefront of the field of regenerative medicine. Based on the minimum criteria stablished by the International Society of Cellular Therapy (ISCT), human MSCs are multipotent progenitor cells that spontaneously adhere to plastic under standard culture conditions; express CD73, CD90, and CD105 cell-surface epitopes and lack CD14 (or CD11b), CD34, CD45, and human leukocytic antigen (HLA)-DR; and are able to differentiate *in vitro* into adipocytes, chondrocytes, and osteoblasts using differentiation protocols (Horwitz et al., 2005; Dominici et al., 2006). MSCs are present in virtually all tissues, but the most common sources used are bone marrow (BM), adipose tissue (AD), and umbilical cord (UC).

Over the past decade, an increasing body of literature has demonstrated the potential of MSCs in promoting therapeutic actions in various experimental models, such as acute respiratory distress syndrome (ARDS) (Németh et al., 2009; Krasnodembskaya et al., 2012; Silva et al., 2018; Lopes-Pacheco et al., 2020), allergic asthma (Goodwin et al., 2011; Lathrop et al., 2014; Malaquias et al., 2018; Castro et al., 2020), COPD/emphysema (Ingenito et al., 2012; Antunes et al., 2014; Poggio et al., 2018; Antunes et al., 2021), idiopathic pulmonary fibrosis (Cargnoni et al., 2012; Cahill et al., 2016; Moroncini et al., 2018), and silicosis (Lopes-Pacheco et al., 2013; Phinney et al., 2015; Lopes-Pacheco et al., 2016; Zhang et al., 2018a). Key therapeutic actions of MSCs include immunomodulation by inhibiting proliferation, maturation, and differentiation of immune cells (de Castro et al., 2019); anti-inflammatory, anti-apoptotic, and antimicrobial activities by secreting paracrine/endocrine mediators (chemokines, cytokines, growth factors, peptides, hormones, lipid mediators, mRNAs, and microRNAs) (Lopes-Pacheco et al., 2020; Lopes-Pacheco et al., 2016; da Silva et al., 2021) or by intercellular contact *via* ligand-receptor recognition and extracellular vesicles (EVs) (Abreu et al., 2021); and wound repair by secreting regenerative factors and transfer of organelles, such as mitochondria (Paliwal et al., 2018; de Carvalho et al., 2021).

The promising therapeutic actions of MSCs in experimental models of respiratory diseases have prompted their assessment in clinical investigations. Although MSC administration was well tolerated and demonstrated a good safety profile, the potential efficacy of MSCs was nevertheless limited (Weiss et al., 2013; Morales et al., 2015; McIntyre et al., 2018; Matthay et al., 2019; Aguiar et al., 2020). Such limited therapeutic effects have been attributed to several factors, including *in vitro* senescence, low number of MSCs infused, functional quiescence or low survival rate of MSCs after the infusion, poor engraftment, and MSC administration in the advanced stage of lung disease. Accordingly, various strategies have been proposed to preserve the stemness of MSCs as well as to functionally potentiate their therapeutic actions. These strategies consist of physical, chemical, and biological methods to condition cells before infusion, and they are based on the biological phenomenon of hormesis, i.e., brief exposure to low doses of an agent known to be harmful at higher doses may lead to beneficial effects (Calabrese et al., 2007). Alternatively, the therapeutic properties of MSCs can be genetically manipulated by overexpressing genes related to cell homing, survival, and immunomodulatory pathways, regardless of exogenous stimuli. In this narrative review, we describe several strategies that are under investigation in the experimental setting for the functional potentiation of the therapeutic properties of MSCs for respiratory diseases. Challenges and future directions are also discussed to efficiently translate MSC-based therapy into the clinical scenario.

## 2 Strategies to potentiate the therapeutic properties of MSCs

**FIGURE 1:**
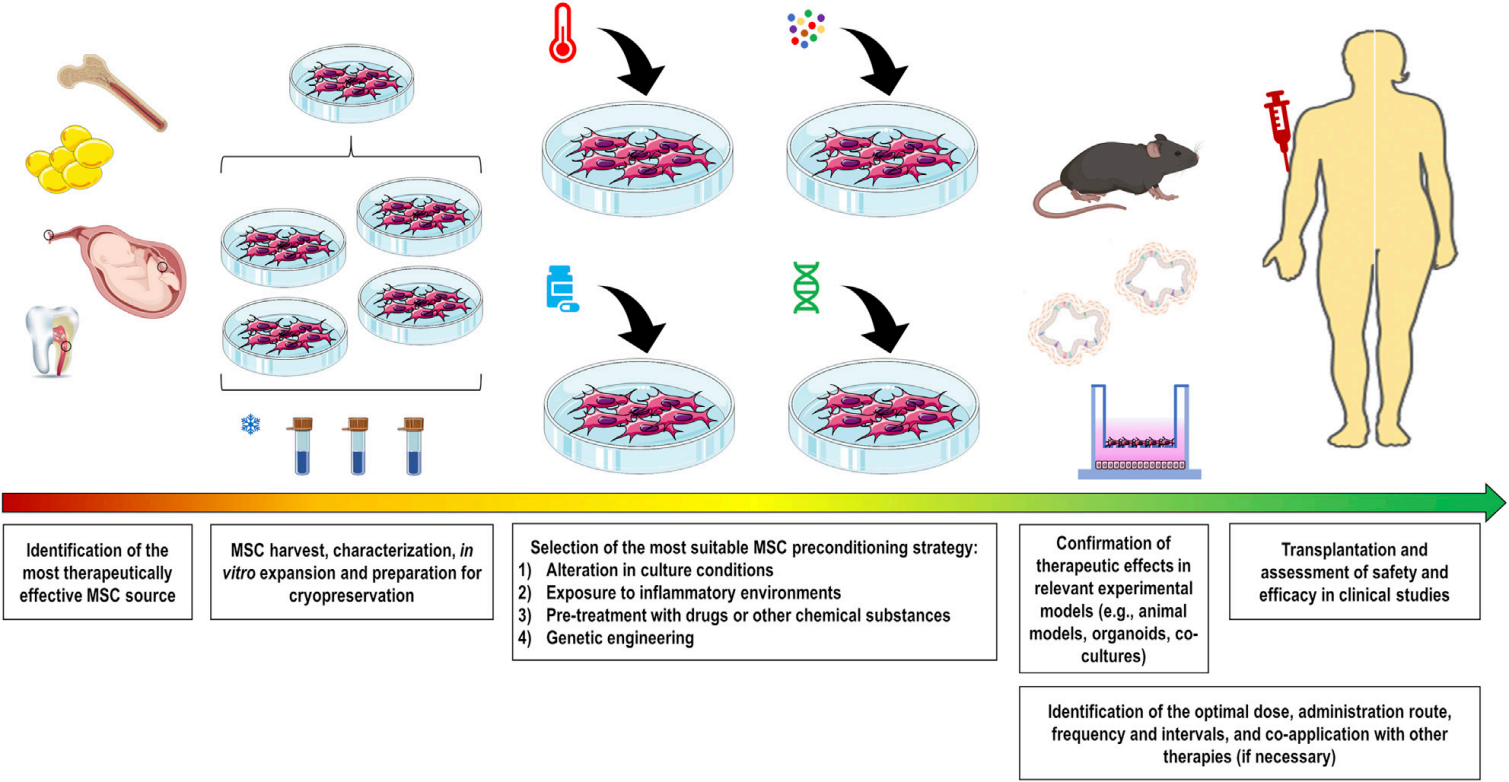
Comprehensive overview showing the production of preconditioned MSCs for pre-clinical and clinical use. Initially, the most therapeutically effective MSC source should be identified for the target disease. After harvesting, cells should be properly characterized and expanded to maximize the number of batches to be frozen. Preconditioning strategy should be selected to functionally potentiate MSC therapeutic properties. These strategies include alteration in culture conditions (O_2_ or CO_2_ concentration, heat shock, nutrient deprivation, and others), exposure to inflammatory conditions (cytokines/chemokines, growth factors, combinations thereof, or biologically relevant samples), pre-treatment with drugs or other substances, and genetic engineering to manipulate expression levels of the gene of interest. The therapeutic abilities of these cells should be validated in relevant experimental models for the subsequent assessment in clinical studies. To enhance the therapeutic outcomes, there are several MSC administration conditions that need to be optimized, including the identification of the optimal dose, administration route, frequency and intervals, and co-application with other therapies if necessary.

Various strategies have been investigated over the past decade to modulate the plasticity of MSCs and thus increase their survival, homing, and therapeutic properties according to the targeted diseases. These strategies include alterations in culture conditions (O_2_ or CO_2_ concentration, heat shock, nutrient deprivation, and others), exposure to inflammatory environments (cytokines/chemokines, growth factors, combinations thereof, or biologically relevant samples), pre-treatment with pharmacological or other chemical molecules, and genetic engineering to manipulate the expression levels of genes of interest (Figure 1).

### 2.1 MSC preconditioning by altering culture conditions

The oxygen availability in MSC niches is significantly variable: 1%–7% in BM, 10%–15% in AD, and ≤5% in the female reproductive tract and birth-related tissues, such as placenta, UC, and Wharton’s jelly (WJ) (Amiri et al., 2015). However, MSCs are usually cultured at a normal atmospheric concentration of oxygen (21% O_2_), which may increase their susceptibility to oxidative stress-induced cellular damage (Boregowda et al., 2012; Amiri et al., 2015; Bétous et al., 2017). When cultured under hypoxia, MSCs exhibited a higher proliferation rate and formed a greater number of colonies compared with those cultured under normoxia (Lennon et al., 2001; Fehrer et al., 2007; Choi et al., 2014; Kwon et al., 2017). Furthermore, hypoxic conditions increased MSC stemness and migration (Das et al., 2012; Saller et al., 2012) and increased the expression of growth factors, such as vascular endothelial growth factor (VEGF), fibroblast growth factor (FGF)-2, and hepatocyte growth factor (HGF) (Choi et al., 2014; Lan et al., 2015; Gorin et al., 2016; Almeria et al., 2019), but reduced the expression of senescence-related β-galactosidase and pro-apoptotic markers (Fehrer et al., 2007; Kwon et al., 2017), as well as lower telomerase shortening rates (Lavrentieva et al., 2010).

Hypoxia-inducible factor (HIF)-1α, a master transcription factor, is stabilized in MSCs under hypoxic conditions (Hu et al., 2008; Li et al., 2017), which promotes a metabolic switch from oxidative phosphorylation to glycolysis and, consequently, decreases generation of reactive oxygen species (ROS) (Estrada et al., 2012; Sart et al., 2014a; Gonzalez-King et al., 2017; Contreras-Lopez et al., 2020). This alteration triggers activation of protein kinase C (PKC), which, in turn, activates nuclear factor kappa B (NF-κB) signaling and upregulates anti-oxidant and anti-apoptotic molecules, such as superoxide dismutase (SOD) and FGF-2 (Otani, 2004; Hu et al., 2008; Afzal et al., 2010). Hypoxia-preconditioned MSCs also demonstrated increased expression of chemokine receptors CXCR4, CXCR7, and CX3CR1, which are associated with the trafficking and homing of infused MSCs to the target organ (Haque et al., 2013). BM-, AD- and UC-MSCs under hypoxia demonstrated enhanced survival by upregulating Bag-1, Bcl-2, and Bcl-X_L_ *via* phosphorylation of AKT (Hu et al., 2008; Bader et al., 2015) and by inhibiting oxidative stress-mediated apoptosis *via* inactivation of caspase-3/-7 activity and lactate dehydrogenase (LDH) release (Bader et al., 2015; Han et al., 2016a). Moreover, the composition of protein and RNA cargo packaged in MSC-derived EVs was substantially modified by hypoxic preconditioning (Gonzalez-King et al., 2017; Yuan et al., 2019; Koch et al., 2022). EVs from hypoxia-preconditioned BM-MSCs demonstrated increased concentration of miR-21, which can regulate cell survival by inhibiting apoptosis and stimulating proliferation (Cui et al., 2018).

Most respiratory diseases cause gas exchange impairment, therefore *in vitro* hypoxic preconditioning may adapt MSCs against stress due to an ischemic environment *in vivo* and increase their survival rate (Table 1). Compared with MSCs under normoxia, hypoxia-preconditioned BM-MSCs demonstrated increased expression of cytoprotective and regenerative factors, leading to greater reduction of interleukin (IL)-1β and IL-6 levels, airway constriction, lung edema, and fibrosis in experimental bleomycin-induced lung fibrosis (Lan et al., 2015). Hypoxia-preconditioned BM-MSCs were also effective at reducing ROS production, apoptosis of lung parenchymal cells and lung fibrosis in experimental radiation-induced lung injury (Li et al., 2017). Therapeutic effects of preconditioning with hypoxia were associated with increased survival of infused BM-MSCs in lung tissue, and upregulation of HIF-1α and AKT as well as SOD and HGF (Lan et al., 2015; Li et al., 2017). In experimental ischemia/reperfusion-induced lung injury, hypoxia-preconditioned BM-MSCs rapidly migrated toward injured lung tissue and reduced cell apoptosis and inflammatory responses related to ROS generation by upregulating glutathione, prostaglandin (PG)E_2_, IL-10 and Bcl-2, and downregulating p38/mitogen-associated protein kinase (MAPK) and the NF-κB signaling pathway (Liu et al., 2017). Moreover, BM-MSCs co-cultured with BM-derived lineage-positive blood cells under hypoxia have been shown to promote proliferation and polarization of macrophages to the M2 anti-inflammatory profile (Takizawa et al., 2017). BM-MSCs have also been shown to be resistant to a higher oxygen concentration (95% O_2_). Administration of conditioned media from hyperoxia-preconditioned BM-MSCs reduced pulmonary hypertension and lung structural abnormalities in a model of hyperoxia-induced neonatal lung injury (Waszak et al., 2012). Such effects were associated with an increase in stannoclacin-1 expression, which has anti-apoptotic actions (Waszak et al., 2012). On the other hand, hypercapnia is another pathologic condition that occurs frequently in chronic respiratory diseases. BM-MSCs cultured in 15% CO_2_ exhibited mitochondrial dysfunction and were unable to promote repair of human primary endothelial or small airway epithelial cells when co-cultured (Fergie et al., 2019), indicating that hypercapnic conditions may significantly hinder the therapeutic properties of MSCs.

**TABLE 1:** *In vivo* preclinical studies assessing the efficacy of MSC preconditioning by altering the culture conditions.

Preconditioning stimulus	MSC source	Model/disease	Delivery route	Regimen	Key findings	Reference
Hypoxia (1.5% O_2_)	BM	C57BL/6 mice, bleomycin-induced lung fibrosis	i.t.	5 × 10^5^ cells, 3 days after injury	↑ HIF-1α, Bcl-2, catalase, HO-1, and VEGF mRNA levels, MSC viability, lung function	Lan et al. (2015)
					↓ lung edema and fibrosis, IL-1β, and IL-6 mRNA levels	
Hypoxia (2.5% O_2_)	BM	C57BL/6 mice, radiation-induced lung injury	i.v.	2 × 10^6^ cells, 2 h after injury	↑ MSC viability and retention in lung tissue, glutathione, and SOD levels	Li et al. (2017)
					↓ lung injury and fibrosis, apoptosis rate, IFN-γ, and TGF-β levels	
Hypoxia (1% O_2_)	BM	Sprague-Dawley rats, ischemia/reperfusion-induced lung injury	i.v.	2.5 × 10^5^ cells, 10 min before the onset of ischemia	↑ glutathione, PGE_2_, and IL-10 levels	Liu et al. (2017)
					↓ lung injury, edema and airway pressure, cell infiltration, MPO activity, IL-1β, MIP-2, and TNF-α levels, apoptosis rate	
Hyperoxia (95% O_2_)	BM	Sprague-Dawley rats, hyperoxia-induced bronchopulmonary dysplasia	i.p.	CM from 1.5 × 10^6^ cells	↑ stanniocalcin-1 levels	Waszak et al. (2012)
					↓ pulmonary arterial hypertension, lung structural abnormalities	
Heat shock (42°C for 1 h)	UC	C57BL/6 mice, LPS-induced pulmonary ARDS	i.n.	1 × 10^4^ cells, 4 h after injury	↑ Hsp70 levels	Lv et al. (2021)
					↓ lung injury and edema, apoptosis rate, BALF cell counts, IL-1β, IL-6, TNF-α, and NLRP3 levels	
96 h serum-free cell culture	AD	Sprague-Dawley rats, CLP-induced polymicrobial sepsis	i.p.	1.2 × 10^6^ cells, 30 min, 6 h, and 18 h after injury	↑ survival rate, 18-h systolic blood pressure, oxygen saturation, glutathione peroxidase, and HO-1 mRNA levels	Chang et al. (2012)
					↓ 72-h AST and creatinine levels, TNF-α, MMP-9, NF-κB, and ICAM-1 mRNA levels	
Hypoxia (1% O_2_) followed by 12 h serum-free cell culture	AD	Sprague-Dawley rats, CLP-induced polymicrobial sepsis	i.p.	1.2 × 10^6^ cells, 30 min, 6 h, and 24 h after injury	↑ glutathione peroxidase and HO-1 levels	Chen et al. (2014)
					↓ circulating T cell populations, white blood cell counts, IL-6 levels, lung injury	
Culture on lung ECM	Lung	Sprague-Dawley rats, ventilator-induced lung injury	i.v.	4 × 10^6^ cells/kg, 30 min after injury	↓ lung elastance, BALF protein concentration, neutrophil and total cell counts, TNF-α, and CXCL2 levels	Nonaka et al. (2020)
3D culture	BM	C57BL/6 mice, bleomycin-induced lung fibrosis	i.n.	10 μg EVs from MSCs, 24 h after final bleomycin challenge	↑ lung fibrosis	Kusuma et al. (2022)
					↓ EV protein content, IDO levels, and macrophage phagocytosis activity	

AD, adipose tissue; ARDS, acute respiratory distress syndrome; AST, aspartate aminotransferase; BALF, bronchoalveolar lavage fluid; BM, bone marrow; CLP, cecal ligation and puncture; CM, conditioned media; EV, extracellular vesicle; HIF, hypoxia-inducible factor; HO, heme oxygenase; Hsp; heat shock protein; ICAM, intercellular adhesion molecule; IDO, indoleamine 2,3-dioxygenase; IFN, interferon; IL, interleukin; i.n., intranasal; i.p., intraperitoneal; i.t., intratracheal; i.v., intravenous; LPS, lipopolysaccharide; MIP, macrophage inflammatory protein; MMP, metalloproteinase; MPO, myeloperoxidase; MSC, mesenchymal stromal cell; NF-κB, nuclear factor κB, PGE, prostaglandin E; SOD, superoxide dismutase; TGF, transforming growth factor; TNF, tumor necrosis factor; UC, umbilical cord; VEGF, vascular endothelial growth factor.

Other strategies that have been investigated to precondition MSCs include heat shock and nutrient deprivation. Compared with normothermic conditions, exposure of BM-MSCs to a higher temperature (38.5°C) induced translocation of heat shock factor 1 (HSF-1) into the nucleus and increased cycloxygenase-2 (Cox-2) expression, which might enhance the immunogenic potential of MSCs (McClain-Caldwell et al., 2018). Heat shock-preconditioned BM-MSCs also induced greater production of IL-10 by co-cultured macrophages (McClain-Caldwell et al., 2018). Furthermore, BM-MSCs under hyperthermia (42°C for 60 min) demonstrated increased expression of heat shock protein (Hsp)27, Hsp70, and Hsp90 (Moloney et al., 2012). These Hsps contribute to cell survival by triggering the phosphoinositide 3-kinase (PI3K)/AKT, extracellular signal-regulated kinases (ERKs), and NF-κB signaling pathways, which upregulate expression of various anti-oxidants, anti-apoptotic and regenerative factors, such as VEGF, FGF-2, HGF, and insulin-like growth factor (IGF)-1 (Sart et al., 2014b). In experimental lipopolysaccharide (LPS)-induced ARDS, heat shock-preconditioned UC-MSCs reduced lung edema, inflammatory cell counts, and levels of inflammation-associated mediators (IL-1β, IL-6, tumor necrosis factor [TNF]-α) (Lv et al., 2021). Such effects were associated with increased Hsp70 levels and inhibition of NLRP3 inflammasome activation in alveolar macrophages (Lv et al., 2021).

MSCs can be induced into a quiescent state by serum depletion, which reduces nucleotide and protein synthesis and other ATP-consuming functions, thus facilitating their survival in ischemic environments (Moya et al., 2017; Ferro et al., 2019). BM-MSC quiescence has been shown to maintain cell viability and function for up 14 days *in vitro* as well as enhance their survival after infusion in mice for up 7 days (Moya et al., 2017). BM-MSC preconditioning with serum depletion inhibits the mammalian target of rapamycin (mTOR), a master regulator of protein translation and proliferation, and stimulates autophagy to protect MSCs against deleterious ischemic conditions. When the autophagic process was pharmacologically inhibited, there was a significant decrease in the survival rate of BM-MSCs under ischemic conditions *in vitro* (Moya et al., 2017). In another study, BM-MSCs were maintained under serum-depleted culture for up 75 days. MSCs transiently altered their morphology and behavior during serum depletion, but maintained their stemness and survived in a quiescent state (Ferro et al., 2019). Preconditioning with serum depletion was found to induce Janus kinase/signal transducer and activator of transcription (Jak/STAT) anti-apoptotic activity, and BM-MSCs used lipid β-oxidation as an alternative energy source (Ferro et al., 2019). On the other hand, AD-MSCs underwent apoptosis by serum depletion, but were shown to increase levels of anti-oxidant factors (glutathione peroxidase and HO-1) and reduce levels of inflammation-related mediators (IL-1β, MIP-1α, and TNF-α), yielding attenuated lung and heart injury and an improved survival rate in experimental cecal ligation and puncture (CLP)-induced sepsis (Chang et al., 2012; Chen et al., 2014).

Although MSCs are often maintained in two-dimensional (2D) monolayer cultures, three-dimensional (3D) culture systems have emerged as an interesting approach, because they may more closely replicate the original physiologic properties of cells and thus enhance their stemness (McKee and Chaudhry, 2017; Deng et al., 2020). The differentiation potential and immunomodulatory properties of MSCs can be influenced not only by soluble bioactive factors and intercellular contact but also by the presence of extracellular matrix (ECM) components, because these elements can control cellular behavior during homeostasis and disease progression (Deng et al., 2020; Raman et al., 2022). Among the supporting materials for 3D cultures (also known as spheroids), hydrogels have been the most commonly used in recent investigations (Murua et al., 2008; McKee and Chaudhry, 2017). Hydrogel encapsulation was able to enhance the viability and proliferation rate of MSCs (Ansari et al., 2017), and chondrogenic (Dvořáková et al., 2014) and osteogenic (Steinmetz et al., 2015) differentiation, as well as promote neovascularization (Chen et al., 2015a) and wound repair (Rustad et al., 2012). AD-MSC spheroids have also been found to have greater levels of pluripotency markers (i.e., CXCR4, Nanog, Sox2, and Oct4), suggesting an increase in MSC stemness in the 3D configuration (Cheng et al., 2013).

Compared with 2D monolayer cultures, MSC spheroids further produced anti-inflammatory and regenerative mediators, including PGE_2_, VEGF, and FGF-2 (Bhang et al., 2011; Ceccaldi et al., 2012; Follin et al., 2016). MSC spheroids also stabilized HIF-1α and upregulated levels of anti-apoptotic markers (e.g., Bcl-X_L_) (Bartosh et al., 2010; Bhang et al., 2011). By using dynamic methods with spinner flasks and a rotating bioreactor, BM-MSC spheroids upregulated IL-24 levels, and their conditioned medium was able to impair the viability of prostate cancer cells (Frith et al., 2010). BM-MSCs were cultured in alginate-based hydrogels with different stiffnesses and then exposed to TNF-α. BM-MSCs preconditioned in the softer hydrogel demonstrated increased clustering of TNF receptors, which led to greater NF-κB activation and downstream responses by exposure to TNF-α (Wong et al., 2020). In a recent study, lung derived MSCs were cultured on lung ECM to biophysically precondition cells in a microenvironment that resembles lung tissue. Administration of preconditioned MSCs led to improved lung elastance and reduced levels of TNF-α and CXCL2 in the bronchoalveolar lavage fluid (BALF) of experimental ventilator-induced lung injury (Nonaka et al., 2020). However, another study reported that 3D culture conditions may hinder certain therapeutic properties of MSCs (Kusuma et al., 2022). Compared with EVs obtained from 2D MSC cultures, BM-MSC spheroid-derived EVs demonstrated reduced anti-inflammatory and anti-fibrotic actions in experimental bleomycin-induced lung fibrosis. Reduced macrophage phagocytosis activity was also observed after *in vitro* incubation with BM-MSC spheroid-derived EVs. Proteomic profiling revealed a significant reduction in the protein content of MSC spheroid-derived EVs and in their global ontology using functional enrichment analysis (Kusuma et al., 2022).

These studies demonstrate that changes in culture conditions potentially have an impact on the therapeutic properties of MSCs. Further studies should be performed to elucidate the potential effects of these preconditioned methods for different respiratory diseases. Moreover, the utility of 3D culture using different supporting materials as a preconditioning method for MSC-based therapies should be further investigated.

### 2.2 MSC preconditioning in an inflammatory environment

There has been increasing interest in preconditioning MSCs in an inflammatory environment by using cytokines/chemokines, growth factors released under pro-inflammatory conditions, or biologically relevant samples from patients, such as BALF and serum (Table 2). Experimental evidence has indicated that MSCs are able to detect the environmental inflammatory signals through their damage- and pathogen-associated surface receptors and tailor their responses according to these stimuli (Waterman et al., 2010; de Castro et al., 2019; Islam et al., 2019). For instance, stimulation of Toll-like receptor (TLR)3 and TLR4 induced generation of regulatory T cells (Treg) in a cell contact-dependent manner (Rashedi et al., 2017). Nevertheless, stimulation of TLR3 and TLR4 led to subsequent activation of different downstream signaling pathways, and MSCs were thus polarized into two different profiles with significantly distinct responses (Waterman et al., 2010). Activation of TLR3 with polyinosinic:polycytidylic acid (poly I:C) led to an anti-inflammatory MSC profile with activation of the Notch signaling pathway and inhibition of T_H_1/T_H_17 cell expansion (Rashedi et al., 2017) as well as production of indoleamine 2,3-dioxygenase (IDO), IL-1RN, IL-4, and PGE_2_ (Waterman et al., 2010; Zhao et al., 2014a; Kim et al., 2018). Poly I:C-preconditioned UC-MSCs also inhibited miR-143 expression and increased production of Cox-2 and macrophage anti-inflammatory activity in experimental CLP-induced sepsis (Zhao et al., 2014a). On the other hand, exposure of MSCs to LPS (a TLR4 agonist) induced a pro-inflammatory MSC profile with upregulation of IL-6, IL-8, and transforming growth factor (TGF)-β (Waterman et al., 2010). Under inflammatory conditions, preconditioning with LPS was found to improve BM-MSC survival (Gupta et al., 2018; Saeedi et al., 2019). Furthermore, when BM-MSCs were harvested from TLR4-deficient mice, they demonstrated impaired survival under *in vitro* inflammatory conditions and were therapeutically inefficient after infusion in an animal model of *Escherichia coli*-induced ARDS (Gupta et al., 2018).

**TABLE 2:** *In vivo* preclinical studies assessing the efficacy of MSC preconditioning in inflammatory conditions.

Preconditioning stimulus	MSC source	Model/disease	Delivery route	Regimen	Key findings	Reference
**Poly I:C (TLR3 agonist)**	UC	C57BL/6 mice, CLP-induced polymicrobial sepsis	i.v.	1 × 10^6^ cells, 1 h after injury	↑ MSC Cox-2, IDO, IL-6, and IL-8 mRNA levels, animal survival rate	Zhao et al. (2014a)
					↓ miR-143 levels, bacterial load, serum CCL5, IL-6, KC, and TNF-α levels	
**LPS (TLR4 agonist)**	BM	Sprague-Dawley rats, *E. coli*-induced sepsis	i.v.	1 × 10^6^ cells, 16 h after injury	↑ LL-37 and hepcidin mRNA levels, mice survival rate	Saeedi et al. (2019)
					↓ bacterial load, circulating lymphocyte counts	
**Pam**_**3**_ **CSK**_**4**_ **(TLR2 agonist)**	BM	Mice, ovalbumin-induced allergic asthma	i.v.	5 × 10^5^ cells, on the same day as ovalbumin challenge	↓ serum IgG levels, BALF IL-4 and IL-5 levels, lung eosinophil counts, muc5ac expression, and airway resistance	Yu and Chiang (2018)
**Flagellin (TLR5 agonist)**	AD	BALB/c mice, LPS-induced extrapulmonary ARDS	i.v.	CM from cells, once for 2 days before injury	↑ macrophage polarization into M2 profile, IL-10 levels	Li et al. (2020)
					↓ IL-1, IL-6, and TNF-α mRNA levels, lung proteinaceous exudate, BALF neutrophil, macrophage and total cell counts, lung injury	
**IFN-γ**	UC	Sprague-Dawley rats, *E. coli*-induced pulmonary ARDS	i.v.	EVs from 3.5–4 × 10^7^ cells, 30 min after injury	↑ rat survival rate, macrophage phagocytosis activity, lung eNOS expression	Varkouhi et al. (2019)
					↓ bacterial load, alveolar-arterial oxygen gradient, BALF proteinaceous exudate and TNF-α levels, lung injury	
**IFN-γ, IL-1β, or IL-12**	BM	C57BL/6 mice, pulmonary ARDS induced by *P. aeruginosa* or *S. aureus*	retro-orbital sinus	1 × 10^6^ cells, 24 h after injury	↑ LL-37 levels	Sutton et al. (2016)
					↓ bacterial load	
**IL-1β**	UC	C57BL/6 mice, CLP-induced polymicrobial sepsis	i.v.	1 × 10^6^ cells or 30 μg EVs, 4 h after injury	↑ mice survival rate, IL-10 levels, macrophage polarization into M2 profile	Song et al. (2017)
					↓ bacterial load, liver, lung and kidney injury, ALT, AST, creatinine, IL-6 and TNF-α levels	
**TGF-β1**	UC	Sprague-Dawley rats, LPS-induced extrapulmonary ARDS	i.v.	5 × 10^5^ cells, 1 h after injury	↓ lung edema, BALF proteinaceous exudate	Li et al. (2016)
**IL-1β/IFN-γ/TNF-α**	BM	Sprague-Dawley rats, ventilator-induced lung injury	i.v.	1 × 10^7^ cells, 6 h after injury	↑ cell viability, PO_2_, static lung compliance, KGF levels	Horie et al. (2020a)
					↓ IL-8 and NF-κB expression, LDH release, lung injury and edema, IL-6 levels	
**Serum and BALF from asthmatic mice**	BM	C57BL/6 mice, HMD-induced allergic asthma	i.t.	1 × 10^5^ cells, 24 h after last HDM challenge	↑ MSC apoptosis, IDO-1, IFN-γ, IL-1RN, IL-10, TSG-6, and TGF-β mRNA expression, macrophage polarization into M2 profile	Abreu et al. (2019a)
					↓ IL-4, IL-13, and eotaxin levels, BALF lymphocyte, macrophage, neutrophil and total cell counts, lung collagen fiber content, static lung elastance	
**Serum from ARDS mice**	BM	C57BL/6 mice, LPS-induced pulmonary and extrapulmonary ARDS	i.v.	1 × 10^5^ cells or EVs, 24 h after injury	MSCs were more effective than EVs regardless of preconditioning with serum	Silva et al. (2019a)
**Serum from injured treated pigs**	BM	Yorkshire pigs, smoke inhalation/burn-induced ARDS	i.v.	∼6 × 10^5^ cells/kg, 30 min after injury	Strong PGE_2_-depdendent immunomodulatory responses	Xu et al. (2019)
					↑ IL-1RN, IL-4, IL-6, IL-10, and IL-13 levels	
					↓ TNF-α levels	
**Serum from ARDS patients**	BM	C57BL/6 mice, LPS-induced extrapulmonary ARDS	i.v.	5 × 10^5^ cells, concomitant to injury	↑ IL-10 levels	Bustos et al. (2013)
					↓ serum IL-1β, IL-6, TNF-α levels, BALF G-CSF, IL-2, and IL-6 levels, lymphocyte, macrophage and neutrophil cell counts, lung edema	
**BALF from ARDS patients**	BM	C57BL/6 mice, LPS-induced pulmonary ARDS	i.n.	2.5 × 10^5^ cells or EVs, 4 h after injury	↑ macrophage polarization into M2 profile	Morrison et al. (2017)
					↓ BALF neutrophil and total cell counts, TNF-α levels, proteinaceous exudate	

AD, adipose tissue; ARDS, acute respiratory distress syndrome; ALT, alanine aminotransferase; AST, aspartate aminotransferase; BALF, bronchoalveolar lavage fluid; BM, bone marrow; CLP, cecal ligation and puncture; Cox-2, cyclooxygenase-2; eNOS, endothelial nitric oxide synthase; EV, extracellular vesicle; G-CSF, granulocyte colony stimulating factor; IDO, indoleamine 2,3-dioxygenase; IFN, interferon; IL, interleukin; i.n., intranasal; i.t., intratracheal; i.v., intravenous; KC, keratinocyte chemoattractant; LDH, lactate dehydrogenase; LPS, lipopolysaccharide; MSC, mesenchymal stromal cell; TGF, transforming growth factor; TLR, Toll-like receptor; TNF, tumor necrosis factor; TSG-6, TNF-stimulated gene 6; UC, umbilical cord.

The microenvironmental conditions of a disease may have a different impact on MSC TLR activation *in vivo*, and MSCs express various TLRs in addition to TLR3 and TLR4 (de Castro et al., 2019). Activation of TLR2 inhibited chemotaxis of BM-MSCs and mitigated MSC-mediated expansion of the Treg population *in vitro* (Lei et al., 2011). In experimental ovalbumin-induced allergic asthma, activation of BM-MSC TLR2 by the agonist Pam_3_CSK_4_ led to a reduction in levels of BALF T_H_2 cytokines (IL-4 and IL-5) and eosinophil counts in lungs, resulting in attenuation of airway resistance in response to incremental doses of methacholine (Yu and Chiang, 2018). Furthermore, activation of UC-MSC TLR5 by flagellin promoted increased expression of CCL24, IL-10, and TGF-β, whereas expression of CCL5 and IP-10 was reduced (Li et al., 2015). Preconditioning with flagellin enhanced the therapeutic actions of AD-MSCs, resulting in a decrease in lung exudate, cell infiltration, and levels of inflammation-associated mediators (IL-1, IL-6, monocyte chemoattractant protein [MCP]-1 and TNF-α) in experimental LPS-induced ARDS (Li et al., 2020). Conditioned media from flagellin-preconditioned MSCs also induced macrophages to polarize into an M2 anti-inflammatory profile (Li et al., 2020).

Under normal physiologic conditions, MSCs exhibit very low expression of major histocompatibility complex (MHC) type I, whereas type II is present intracellularly but absent on the cell surface (Le Blanc et al., 2003). Upon interferon (IFN)-γ stimulation, MHC class II and costimulatory factor (CD40, CD80, and CD86) can traffic to the cell surface, but MSCs are still able to escape recognition by alloreactive T cells and displayed enhanced immunomodulatory activities (Le Blanc et al., 2003; Ankrum et al., 2014; De Witte et al., 2016). Proteomic analysis revealed that preconditioning with IFN-γ altered BM-MSC expression of over 200 proteins, of which 169 were upregulated and 41 were downregulated (Guan et al., 2017). Exposure of MSCs to IFN-γ led to upregulation of IDO (De Witte et al., 2016; Wang et al., 2016; Guan et al., 2017), several immunomodulatory mediators (CCL2, HGF, PGE_2_, and TFG-β) (De Witte et al., 2016), adhesion molecules (ICAM-1 and VCAM-1) (Wang et al., 2016; Guan et al., 2017) and chemokines (CXCL9, CXCL10, CXCL11) (Wang et al., 2016), and promoted inhibition of NK cell activation (Noone et al., 2013) and immunosuppressive effects on T lymphocytes (Krampera et al., 2006). When IFN-γ blocking antibody was used, BM-MSC-induced immunosuppression was abrogated (Krampera et al., 2006). The protective effect of BM-MSCs against ovalbumin-induced allergic asthma was also abrogated when cells were infused in IFN-γ null mice (Goodwin et al., 2011). In another study, preconditioning with IFN-γ inhibited mTOR signaling by early phosphorylation of STAT1 and STAT3, increasing the ability of BM-MSCs to suppress T cell proliferation (Vigo et al., 2017). IFN-γ-preconditioned BM-MSCs were also effective at inhibiting T cell proliferation and subsequent secretion of T_H_1 cytokines by upregulating programmed cell death-1 ligands (PDL-1), regardless of IDO upregulation (Chinnadurai et al., 2014). Furthermore, when co-cultured with activated lymphocytes, IFN-γ-preconditioned MSCs reduced T_H_17 cell counts and production of IFN-γ and TNF-α (Wang et al., 2016). Although preconditioning with IFN-γ also led to increased expression of MHC type I, it significantly reduced the susceptibility of UC-MSCs to NK cytotoxicity by reducing the cell surface expression of NKG2D (Noone et al., 2013). In experimental *E. coli*-induced ARDS, EVs from both naive and IFN-γ-preconditioned UC-MSCs were similarly able to reduce mortality and improve bacterial killing and phagocytosis (Varkouhi et al., 2019). However, EVs from IFN-γ-preconditioned UC-MSCs were more effective at reducing alveolar protein leak, TNF-α levels, and alveolar-arterial oxygen gradient but enhanced production of endothelial nitric oxide synthase (eNOS) (Varkouhi et al., 2019).

In addition to IFN-γ, TNF-α, IL-1β, and IL-17 have been identified as key inflammatory cytokines to enhance the therapeutic properties of MSCs before infusion. Preconditioning with TNF-α promoted upregulation of several immunomodulatory factors in MSCs, including IDO, HGF, PGE_2_, and TNF-α-stimulated gene 6 (TSG-6), although to a lesser extent in comparison with MSC preconditioning with IFN-γ (English et al., 2007; Prasanna et al., 2010; De Witte et al., 2016). Nevertheless, preconditioning with TNF-α had superior effects on migration of BM- and UC-MSCs and secretion of IL-8 in comparison with preconditioning with IFN-γ (Hemeda et al., 2010). In experimental CLP-induced sepsis, infusion of BM-MSCs exposed to TNF-α neutralizing antibody or harvested from TNF-R1 knockout mice was unable to protect against the deleterious effects of sepsis (Németh et al., 2009). Conditioned media from TNF-α-preconditioned BM-MSCs induced Cox-2/PGE_2_ signaling activation and led to inhibition of B-cell IgE production and histamine release, thus alleviating allergic symptoms (Su et al., 2015). On the other hand, global transcriptome profiling of IL-1β-preconditioned BM-MSCs revealed upregulation of several genes related to NF-κB signaling and its downstream responses, namely, cell survival, migration, cytokine production, angiogenesis, and immune responses (Carrero et al., 2012). IL-1β-preconditioned UC-MSCs demonstrated higher migration to inflammatory foci and led to increased percentages of Treg and T_H_2 cells and decreased T_H_1 and T_H_17 cell counts in mesenteric lymph nodes and spleen (Fan et al., 2012). BM-MSCs preconditioned with IL-1β, IL-12, or IFN-γ also demonstrated increased secretion of the antimicrobial peptide LL-37, thus significantly reducing the rate of growth of *Pseudomonas aeruginosa*, *Staphylococcus aureus*, and *Streptococcus pneumonia* (Sutton et al., 2016). Furthermore, preconditioning with IL-1β was reported to potentiate gingival derived-MSC immunomodulatory and wound repair properties by upregulating expression of TGF-β and matrix metalloproteinases (MMPs) (Magne et al., 2020). In another study, IL-1β-preconditioned UC-MSCs produced EVs with high levels of miR-146a, which resulted in M2 polarization when transferred to BM-derived macrophages *in vitro* (Song et al., 2017)*.* Administration of IL-1β-preconditioned UC-MSCs led to decreased serum levels of IL-6, TNF-α, alanine aminotransferase and aspartate aminotransferase, and liver, lung, and kidney injury and increased bacterial clearance and survival in experimental CLP-induced sepsis (Song et al., 2017).

Differentiation and proliferation of MSCs have been modulated by preconditioning with IL-17 in a dose-dependent manner (Huang et al., 2009; Sivanathan et al., 2015). These effects were associated with the generation of ROS from stimulation of TNF receptor-associated factor 6 (TRAF-6) and the Act-1 adaptor that activated the MEK/ERK signaling pathway (Huang et al., 2009). MSC preconditioning with IL-17 also promoted greater osteogenic differentiation and MSC migration by increasing expression of CXCL6, MMP1, and MMP13 (Huang et al., 2009; Noh, 2012; Sivanathan et al., 2015) and inhibited adipogenic differentiation by upregulating IL-6 and IL-8 upon differentiation (Shin et al., 2009). The immunosuppressive potential of BM-MSCs was enhanced by preconditioning with IL-17, leading to inhibition of effector T cell proliferation and decreased secretion of T_H_1 cytokines (IFN-γ, IL-2, and TNF-α) and promoted Treg cell expansion (Sivanathan et al., 2015; Sivanathan et al., 2017). Furthermore, IL-17-preconditioned BM-MSCs demonstrated expression of MHC class I, II and costimulatory molecules comparable with that of naive MSCs, indicating they maintained a hypoimmunogenic profile (Sivanathan et al., 2017).

Growth factors have also been considered as preconditioning factors to enhance the therapeutic properties of MSCs. In this context, preconditioning with TGF-β promoted BM-MSC mobilization and migration during bone remodeling and facilitated peripheral tissue healing *via* the non-canonical Smad-independent signaling pathway (Dubon et al., 2018). TGF-β-preconditioned UC-MSCs demonstrated an extended period of survival in the lungs of experimental LPS-induced ARDS, and reduced lung edema and BALF neutrophil counts (Li et al., 2016). Furthermore, preconditioning with FGF-2 enhanced the angiogenic properties of dental pulp-derived MSCs by increasing expression of HGF and VEGF more efficiently than hypoxia preconditioning (Gorin et al., 2016). In addition to a single cytokine or growth factor, preconditioning with a cocktail of inflammatory mediators has been investigated. For instance, IFN-γ and TNF-α were found to work synergistically in enhancing the immunomodulatory properties of MSCs. MSC preconditioning with both cytokines led to inhibition of complement activation by increasing the production of factor H in a time- and dose-dependent manner (Tu et al., 2010). IFN-γ and TNF-α promoted chromatin remodeling closer to the transcriptional start site for IDO1, which remained altered even during cryopreservation (Gonzalez et al., 2016). After thawing, previously preconditioned BM-MSCs demonstrated quick accumulation of high levels of IDO1 mRNA upon re-exposure to these cytokines (Gonzalez et al., 2016). BM-MSC preconditioning with IFN-γ/TNF-α increased IDO activity, which promoted monocyte differentiation into M2 anti-inflammatory macrophages, which, in turn, induced suppression of T cell proliferation (François et al., 2012a). Exposure of BM-MSCs to IFN-γ/TNF-α led to secretion of PD-1 ligands (PD-L1 and PD-L2), which downregulated IL-2 and suppressed CD4^+^ T cell activation, inducing immunosuppression by irreversible hyporesponsiveness and subsequent apoptosis (Davies et al., 2017). In another study, inhibition of T cell proliferation by IFN-γ/TNF-α-preconditioned BM-MSCs was associated with upregulation of NOS2 and subsequent increase in NO generation as well as attenuation of delayed hypersensitivity reactions (Szabó et al., 2015).

Several other cocktails of inflammatory mediators have been used to precondition MSCs. Compared with preconditioning with IFN-γ/TNFα, exposure of nasal mucosa-derived MSCs to IL-1β/IFN-γ/TNFα enhanced neutrophilia by further increasing IL-8 secretion in a mechanism mediated by activation of STAT5 and p38/MAPK signaling (Hackel et al., 2021). On the other hand, IL-1β/IFN-γ/TNFα-preconditioned BM-MSCs reduced structural abnormalities and inflammation in lungs and restored oxygenation and improved lung compliance in experimental ventilator-induced lung injury (Horie et al., 2020a). The enhanced epithelial wound repair was mediated in part by keratinocyte growth factor (KGF) secretion (Horie et al., 2020a). In another study, IL-1β/TNFα-preconditioned BM-MSCs prolonged graft survival by stimulating lung-derived myeloid cells to inhibit lymphocyte proliferation and promote Treg cell expansion (Murphy et al., 2019). Immunosuppressive actions of BM-MSCs were also remarkably increased by preconditioning with IL-17/IFN-γ/TNF-α in an inducible NO synthase (iNOS)-induced mechanism (Han et al., 2014). LPS and TNF-α acted synergistically and increased arginase-1 and PGE_2_ secretion by preconditioned MSCs. LPS/TNF-α-preconditioned BM-MSCs also induced macrophage polarization *in vitro* to an M2 profile and improved osteogenesis (Croes et al., 2015; Lin et al., 2017). IL-1β/IL-6/IL-23-preconditioned AD- and BM-MSCs displayed no changes in morphology, immunophenotype, and costimulatory factors, with an exception for upregulation of CD45. Furthermore, MSC immunomodulatory activity was well preserved with increased IL-10 secretion and decreased IL-4 after preconditioning with IL-1β/IL-6/IL-23 (Pourgholaminejad et al., 2016).

To more closely replicate lung inflammatory milieu, BALF and serum from animal models or patients with inflammatory lung conditions have been used as surrogates in MSC preconditioning. Exposure of MSCs to BALF from patients with cystic fibrosis (CF) infected with *Aspergillus* sp. induced rapid MSC apoptosis partly related to the presence of fungal-produced gliotoxin, which led to mitochondrial dysfunction (Abreu et al., 2020). RNA analysis revealed differential expression of transcripts involved in IFN signaling, antimicrobial activity, and cell death by MSCs exposed to CF BALF positive versus negative to *Aspergillus* infection (Abreu et al., 2020). BM-MSCs were also induced to undergo apoptosis by exposure to either BALF or serum from experimental house dust mite (HDM)-induced allergic asthma, and this process increased expression of immunomodulatory mediators (IDO-1, IFN-γ, IL-1RN, IL-10, TSG-6, and TGF-β) (Abreu et al., 2019a). Serum was more effective than BALF in preconditioning BM-MSCs and led to a significant reduction in lung inflammatory cell counts and levels of T_H_2 cytokines (IL-4 and IL-13) and eotaxin, and lung function improved in a murine model of HDM-induced allergic asthma (Abreu et al., 2019a).

BM-MSCs were preconditioned with serum from experimental LPS-induced ARDS and their EVs were harvested. Regardless of preconditioning with serum, MSCs and EVs were able to reduce levels of inflammation-related mediators (IL-6, keratinocyte chemoattractant [KC], TGF-β, TNF-α), alveolar collapse, and BALF inflammatory cell counts (Silva et al., 2019a). Nevertheless, MSCs were more effective than EVs in reducing lung edema and fibrosis, resulting in improved lung function in both experimental pulmonary and extrapulmonary ARDS (Silva et al., 2019a). A significant immunomodulatory response in a PGE_2_-dependent mechanism was observed when BM-MSCs were reconditioned with serum from porcine smoke inhalation/burn-induced ARDS treated with MSCs (Xu et al., 2019). BM-MSCs were also preconditioned with serum from patients with moderate to severe ARDS, which contained high levels of IL-6, IL-8, and IL-10 (Bustos et al., 2013). After preconditioning with serum, BM-MSCs demonstrated enhanced expression of IL-1RN and IL-10 *in vitro* and significantly reduced BALF inflammatory cell counts, lung injury, and vascular permeability in experimental LPS-induced ARDS (Bustos et al., 2013).

BALF from either patients with ARDS or health controls (HC) demonstrated no cytotoxicity to MSCs, but they clearly modulated MSC expression of various pro- and anti-inflammatory mediators (Enes et al., 2021). IL-1β in HC and ARDS BALF was predictive of MSC production of certain pro-inflammatory mediators, such as IL-6 and IL-8. MSCs exposed to HC BALF, but not to ARDS BALF, demonstrated increased expression of HLA class II factors, suggesting that MSCs can be stimulated by normal lung milieu to cooperate in immune surveillance (Enes et al., 2021). Another study demonstrated that preconditioning with ARDS BALF promoted anti-inflammatory actions of BM-MSCs and *in vitro* macrophage phagocytosis activity (Morrison et al., 2017). Nevertheless, conditioned media from ARDS BALF-preconditioned BM-MSCs were less effective in promoting monocyte polarization to an anti-inflammatory profile than conditioned media from CF BALF-preconditioned MSCs (Abreu et al., 2019b).

These studies demonstrate that disease environmental conditions have a major impact on MSC survival and immunomodulatory activities, and the microenvironments in certain diseases (or sub-phenotypes) may be more suited to facilitating MSC activation and promoting optimal MSC therapeutic actions.

### 2.3 MSC preconditioning with pharmacological agents or other substances

In preclinical research, MSCs have been preconditioned with a range of drugs or other chemical agents not only to enhance their therapeutic properties and homing but also to improve their survival rate and resistance against any harmful stimuli they may encounter (Table 3). For instance, exposure of MSCs to sub-lethal doses of H_2_O_2_ (≤50 µM) was shown to increase their resistance against a lethal concentration of this chemical agent (Sart et al., 2014b). BM-MSCs exposed to low doses of H_2_O_2_ exhibited increased expression of CXCR4 and migration-mediated stromal cell-derived factor (SDF)-1α (Li et al., 2009). H_2_O_2_-preconditioned BM-MSCs were also protected against apoptosis induced by a higher concentration of H_2_O_2_ (500 µM) due to activation of the ERK pathway, which upregulated anti-apoptotic factors such as Bcl-2 and Bcl-X_L_ (Li et al., 2009; Sart et al., 2014b). In AD-MSCs, long-term exposure to a low dose (10 µM) of H_2_O_2_ led to increased survival by upregulating nuclear factor-erythroid 2-related factor 2 (Nrf2) and several anti-oxidant mediators (catalase, glutathione peroxidase-1, heme oxygenase (HO)-1 and SOD) but reduced intracellular ROS levels and expression of Cox-2 and IL-1β (Garrido-Pascual et al., 2020). Administration of H_2_O_2_-preconditioned UC-MSCs reduced lung remodeling by attenuating expression of α-smooth muscle actin (α-SMA) and TGF-β as well as myeloperoxidase (MPO) activity in experimental bleomycin-induced lung fibrosis (Mahmoudi et al., 2020). Preconditioning with pyrogallol was also able to enhance UC-MSC therapeutic properties *via* activation of the Nrf2/HO-1 signaling pathway, leading to a reduction of lung injury, epithelial cell apoptosis, MPO activity, and levels of inflammation-associated mediators (IL-6, IL-8, MCP-1, and TNF-α) in experimental LPS-induced ARDS (Zhang et al., 2022).

**TABLE 3:** *In vivo* preclinical studies assessing the efficacy of MSC preconditioning with pharmacological agents or other substances.

Preconditioning stimulus	MSC source	Model/Disease	Delivery route	Regimen	Key findings	Reference
**H**_**2**_ **O**_**2**_	UC	C57BL/6 mice, bleomycin-induced lung fibrosis	i.t.	2 × 10^5^ cells, 7 days after injury	↑ normal alveolar space	Mahmoudi et al. (2020)
					↓ lung fibrosis, TGF-β1 and α-SMA expression, MPO activity	
**Pyrogallol**	UC	Sprague-Dawley rats, LPS-induced pulmonary ARDS	i.v.	1 × 10^7^ cells, 2 h before LPS challenge	↑ Bcl-2 expression, Nrf2 and HO-1 levels	Zhang et al. (2022)
					↓ lung injury, apoptosis rate and Bax expression, MPO activity, IL-6, IL-8, MCP-1, and TNF-α levels	
**ATRA**	BM	C57BL/6 mice, elastase-induced emphysema	i.v.	2 × 10^5^ cells, 21 days after injury	↑ lung static elastance, MSC retention	Takeda et al. (2018)
					↓ alveolar hyperinflation	
**Pioglitazone**	AD	C57BL/6 mice, cigarette smoke-induced emphysema	i.t.	1 × 10^5^ cells, 6 months after injury	↑ lung epithelial cell proliferation, FGF-2, HGF, and VEGF levels	Hong et al. (2016)
					↓ alveolar hyperinflation, caspase-3/-7 activity	
**Oncostatin M**	BM	C57BL/6 mice, bleomycin-induced lung fibrosis	i.t.	2 × 10^5^ cells, 3 days after injury	↑ HGF mRNA levels, wound closure, lung function	Lan et al. (2017)
					↓ fibronectin, collagen I, IL-1β and IL-6 mRNA levels, lung edema and fibrosis, BALF neutrophil and total cell counts	
***N*-Acetylcysteine**	Embryonic	BALB/c, bleomycin-induced lung fibrosis	i.v.	2 × 10^5^ cells, 24 h after injury	↑ mice survival rate	Wang et al. (2013a)
					↓ lung injury and fibrosis, BALF macrophage, neutrophil and lymphocyte counts, IL-1β, IL-6, and TNF-α levels	
**EPA**	BM	C57BL/6 mice, HMD-induced allergic asthma	i.t.	1 × 10^5^ cells, 24 h after last HDM challenge	↑RvD_1_, PGE_2_, IL-10, and TGF-β levels, lung function	Abreu et al. (2018)
					↓ BALF IL-4, IL-13, and VEGF levels, eosinophil, macrophage, lymphocyte and neutrophil cell counts, alveolar collapse, airway resistance, lung fibrosis, and mucus-producing cell counts	
**EPA**	AD	C57BL/6 mice, CLP-induced polymicrobial sepsis	i.v.	1 × 10^5^ cells, 24 h after injury	↑ RvD_1_, PGE_2_, IL-10, and TGF-β levels, mice survival rate, lung function, VEGF levels	Silva et al. (2019b)
					↓ sepsis severity score, lung inflammation, fibrosis and edema, alveolar collapse, IL-1β and KC levels, distal organ injury	

AD, adipose tissue; ARDS, acute respiratory distress syndrome; ATRA, all-*trans* retinoic acid; BALF, bronchoalveolar lavage fluid; BM, bone marrow; CLP, cecal ligation and puncture; EPA, eicosapentaenoic acid; HGF, hepatocyte growth factor; HO, heme oxygenase; IL, interleukin; i.t., intratracheal; i.v., intravenous; KC, keratinocyte chemoattractant; LPS, lipopolysaccharide; MCP, monocyte chemoattractant protein; MPO, myeloperoxidase; MSC, mesenchymal stromal cell; Nrf2; nuclear factor-erythroid 2-related factor 2; RvD, resolvin D; SMA, smooth muscle actin; TGF, transforming growth factor; TNF, tumor necrosis factor; UC, umbilical cord; VEGF, vascular endothelial growth factor.

Although MSCs are subjected to the lung first-pass effect and are thus easily trapped in pulmonary capillaries after systemic infusion (Fischer et al., 2009), their retention in lung tissue rarely exceeds a few days (Leblond et al., 2009; Wang et al., 2009; De Oliveira et al., 2017; de Witte et al., 2018). As MSC engraftment is primarily mediated by the interaction between CXCR4 and SDF-1 (Jin et al., 2018), pharmacological modulation of these may enable improved MSC homing to injured tissues. UC-MSC preconditioning with low doses of valproic acid, a histone deacetylase inhibitor, enhanced MSC recruitment to injury sites by upregulating CXCR4 and increased MSC anti-inflammatory responses (Marquez-Curtis et al., 2014; Lim et al., 2017). Preconditioning with rapamycin, ethionamide, or the DNA methyltransferase inhibitor, 5-azacytidine, was able to increase MSC CXCR4 expression, thus improving the migration ability of UC- and WJ-MSCs (Lee et al., 2015; Zheng et al., 2019; Lee et al., 2020). When co-cultured with activated T cells, rapamycin- or 5-azacytidine-preconditioned MSCs demonstrated immunosuppressive properties by upregulating Cox-2 and PGE_2_ (Lee et al., 2015; Wang et al., 2017). BM-MSC homing and CXCR4 expression were also increased by resveratrol treatment before preconditioning with SDF-1α (Hajinejad et al., 2018). In addition to these compounds, screening of ∼9000 signal-transduction modulators was performed to identify novel compounds able to enhance MSC surface expression of homing ligands (Levy et al., 2015). In this context, preconditioning with Ro-31-8425 enabled the *in vivo* delivery BM-MSCs to inflammatory sites in a CD11a-dependent mechanism (Levy et al., 2015).

Desferrioxamine and 2,4-dinitrophenol, two hypoxia-mimetic agents, were able to enhance MSC homing and immunomodulatory actions (Najafi and Sharifi, 2013; Khan et al., 2016). BM-MSCs preconditioned with 2,4-dinitrophenol exhibited increased expression of genes related to cell adhesion and angiogenesis (Khan et al., 2016); preconditioning of BM-MSCs with low doses of desferrioxamine led to stabilization of HIF-1α by preventing its hydroxylation, decreased mitochondrial activity and apoptosis, and upregulation of glycolysis-related genes (Fujisawa et al., 2018). Short-term exposure of BM-MSCs to the volatile anesthetic isoflurane also increased expression of HIF-1α, CXCR4, and SDF-1 (Sun et al., 2015). On the other hand, exposure of BM-MSCs to curcumin, a natural dietary product, and subsequent hypoxia improved cell survival and promoted mitochondria fusion (Wang et al., 2020). Preconditioning with curcumin/hypoxia suppressed BM-MSC apoptosis by mitigating cytochrome c release from the mitochondria and caspase-3 cleavage (Wang et al., 2020). Moreover, curcumin/hypoxia-preconditioned BM-MSCs accelerated wound repair *in vivo* (Wang et al., 2020). Although these preconditioning strategies led to more MSCs moving into and remaining in certain injured tissues longer, their efficacy for respiratory diseases has yet to be assessed.

All-*trans* retinoic acid (ATRA) regulates transcription of genes related to apoptosis, differentiation, and immune responses. When BM-MSCs were preconditioned with ATRA, there was increased expression of Cox-2, CXCR4, CCR2, HIF-1, angiopoietin (Ang)-2, and Ang-4, which was abrogated by Cox-2 inhibition (Pourgholaminejad et al., 2016). BM-MSCs preconditioned with ATRA also demonstrated enhanced potential of wound repair *in vivo* (Pourgholaminejad et al., 2016)*.* In this context, preconditioning with ATRA promoted *in vitro* activation of BM-MSC p70S6 kinase-1 and led to significant improvements in lung structure (mean linear intercepts and alveolar surface area) and function (static lung compliance) after infusion of ATRA-preconditioned MSCs in experimental elastase-induced emphysema (Takeda et al., 2018). AD-MSCs preconditioned with pioglitazone, an antidiabetic drug that binds to peroxisome proliferator-activated receptor (PPAR)-γ, also enhanced lung tissue repair and upregulated VEGF *in vitro* (Hong et al., 2016). In experimental emphysema induced by cigarette smoke, administration of pioglitazone-preconditioned AD-MSCs led to mitigation of lung structure abnormalities measured by mean linear intercepts and increased expression of several growth factors (FGF-2, HGF, and VEGF) (Hong et al., 2016).

Screening of a library containing over 1400 bioactive compounds approved by the US Food and Drug Administration identified tetrandrine, a calcium channel inhibitor, as a potential hit for MSC preconditioning. *In vitro* exposure of BM-MSCs to tetrandrine increased expression of PGE_2_ by the NF-κB/Cox-2 signaling pathway and attenuated TNF-α secretion by LPS-activated macrophages (Yang et al., 2016). Furthermore, preconditioning with oncostatin M was effective in promoting BM-MSC migration and increasing HGF expression (Lan et al., 2017). BM-MSCs preconditioned with oncostatin M improved lung function and reduced lung edema, inflammatory cell counts, and fibrosis in bleomycin-induced lung fibrosis and reduced mRNA expression of fibronectin and type I collagen in co-cultured fibroblasts (Lan et al., 2017). The anti-oxidant ability of MSCs preconditioned with the mucolytic agent *N-*acetylcysteine improved *in vitro*, leading to increased cellular glutathione levels and attenuated ROS generation (Wang et al., 2013a). In experimental bleomycin-induced lung fibrosis, MSCs preconditioned with *N-*acetylcysteine reduced lung inflammation and fibrosis, BALF inflammatory cell counts, and levels of inflammation-associated mediators (IL-1β, IL-6, and TNF-α), which resulted in a significant improvement in the survival rate (Wang et al., 2013a).

The omega-3 fatty acid eicosapentaenoic acid (EPA) is another agent that can improve the therapeutic properties of MSCs. Preconditioning with EPA increased the formation of lipid bodies *in vitro* and secretion of resolvin-D_1_, PGE_2_, IL-10, and TGF-β by AD- and BM-MSCs without affecting cell viability (Abreu et al., 2018; Silva et al., 2019b). BM-MSCs preconditioned with EPA decreased BALF inflammatory cell counts, levels of T_H_2 cytokines (IL-4 and IL-13), lung fibrosis, and the presence of mucus-producing cells, and improved lung function in experimental HDM-induced allergic asthma (Abreu et al., 2018). Administration of MSCs preconditioned with EPA also promoted polarization of lung macrophages to an M2 anti-inflammatory profile (Abreu et al., 2018). In experimental CLP-induced sepsis, preconditioning of AD-MSCs with EPA led to a reduction of lung inflammation, edema and alveolar collapse, and levels of IL-1β, KC, and TGF-β, and increased VEGF levels and improved lung function (Silva et al., 2019b). Moreover, administration of AD-MSCs preconditioned with EPA reduced tissue injury not only in lungs but also in distal organs, thus resulting in a decrease in sepsis severity score and improvement in the survival rate (Silva et al., 2019b).

Overall, MSC preconditioning with drugs and other substances is an interesting approach and has been demonstrated to enhance MSC homing and immunomodulatory actions. However, there are still limited data on MSC preconditioning with each drug for respiratory diseases, and further studies are warranted.

### 2.4 Potentiation of MSC therapeutic properties by genetic engineering

Genetic engineering has been used extensively in experimental research not only to enhance the beneficial effects of therapies but also to unravel their mechanism of action by overexpressing or knocking down genes of interest. In MSC-based therapy, several genes related to survival, engraftment, and immunomodulatory actions have been targeted for enhanced and sustained expression to potentiate the therapeutic properties of MSCs (Table 4).

**TABLE 4:** *In vivo* preclinical studies assessing the efficacy of genetically engineered MSCs.

Gene manipulated	MSC source	Model/Disease	Delivery route	Regimen	Key findings	Reference
CXCR4	BM	Sprague-Dawley rats, LPS-induced extrapulmonary ARDS	i.v.	1 × 10^6^ cells, 1 h after injury	↑ MSC migration and homing in lung tissue, IL-10 levels	Yang et al. (2015)
					↓ BALF neutrophil counts, IL-6 and TNF-α levels, lung injury and edema	
EP2	BM	C57BL/6 mice, LPS-induced pulmonary ARDS	i.v.	5 × 10^5^ cells, 4 h after injury	↑ MSC retention in lung tissue, IL-10 levels, mice survival rate	Han et al. (2016b)
					↓ lung injury and edema, IL-1β and TNF-α levels	
ROR2	BM	C57BL/6 mice, LPS-induced pulmonary ARDS	i.t.	5 × 10^5^ cells, 4 h after injury	↑ MSC retention in lung tissue, KGF and IL-10 levels	Cai et al. (2016)
					↓ lung injury and fibrosis, BALF proteinaceous exudate, IL-1β and IL-6 levels	
HO-1	BM	FVB/n mice, hypoxia-induced pulmonary arterial hypertension	i.v.	5 weeks after injury	↑ IL-10 levels	Liang et al. (2011)
					↓ right ventricular systolic pressure, lung vascular remodeling, CCL2 and IL-6 levels	
HO-1	BM	Wistar rats, LPS-induced pulmonary ARDS	i.v.	1 × 10^6^ cells, 2 h after injury	↑ animal survival rate, HGF, KGF, and IL-10 levels, MSC retention in lung tissue	Chen et al. (2019)
					↓ lung injury and edema, BALF neutrophil and total cell counts, IL-1β and TNF-α levels, TLR4, MyD88, and TRIP levels	
Nrf2	Amniotic membrane	C57BL/6 mice, LPS-induced pulmonary ARDS	i.v.	1 × 10^6^ cells, 4 h after injury	↑ IL-10 levels, MSC retention in lung tissue	Zhang et al. (2018b)
					↓ lung injury, edema and fibrosis, epithelial cell apoptosis, IL-1β and IL-6 levels	
SOD	BM	Non-obese diabetic/severe combined immunodeficiency mice, radiation-induced lung injury	i.v.	1 × 10^6^ cells, 4 h after injury	↑ animal survival rate, IL-10 levels	Chen et al. (2017a)
					↓ lung injury, edema and fibrosis, epithelial cell apoptosis, IL-1β, IL-6, TNF-α, TGF-β, and MDA levels	
IL-10	BM	C57BL/6 mice, LPS-induced pulmonary ARDS	i.t.	1 × 10^6^ cells, 4 h after injury	↑ animal survival rate	Wang et al. (2018a)
					↓ BALF proteinaceous exudate and TNF levels	
IL-10	UC	Sprague-Dawley rats, *E. coli*-induced pulmonary ARDS	i.v.	1 × 10^7^ cells, 1 h after injury	↑ animal survival rate, static lung compliance, macrophage phagocytosis activity	Jerkic et al. (2019)
					↓ BALF proteinaceous exudate, bacterial load, IL-6, MCP-1, and TNF-α levels, lung injury	
IL-10 and/or HGF	BM	C57BL/6 mice, HCl-induced lung injury	i.t. and i.v.	1 × 10^6^ cells, 5 min after injury	↓ lung inflammation and injury, IL-6, TNF-α, IL-1β, MCP-1, fibronectin, and fibrinogen levels	Islam et al. (2019)
IL-1RL1	AD	BALB/c mice, LPS-induced pulmonary ARDS	i.v.	1 × 10^6^ cells, 6 h after injury	↑ IL-10 mRNA levels	Martínez-González et al. (2013)
					↓ IL-33, TLR4, IL-1β, and IFN-γ mRNA levels, BALF neutrophil counts and proteinaceous exudate, lung injury	
Del-1	BM	C57BL/6 mice, LPS-induced extrapulmonary ARDS	i.v.	5 × 10^6^ cells, 1 h after injury	↓ lung injury and edema, neutrophil counts, IL-6 and TNF-α levels, MPO activity	Zhao et al. (2014b)
ACE2	UC	C57BL/6 mice, bleomycin-induced lung fibrosis			↓ lung injury and fibrosis, IL-1, IL-6, IFN-γ, TNF-α, and TGF-β levels	Min et al. (2015)
ACE2	BM	C57BL/6 mice, LPS-induced pulmonary ARDS	i.v.	5 × 10^5^ cells, 4 h after injury	↑ MSC retention in lung tissue, BALF IL-10 levels	He et al. (2015)
					↓ lung injury, vascular permeability, angiotensin II expression, BALF neutrophil and total cell counts, IL-1β, IL-6, and iNOS levels	
Ang-1	BM	C57BL/6 mice, LPS-induced pulmonary ARDS	i.v.	2.5 × 10^5^ cells, 30 min after injury	↓ BALF neutrophil and total cell counts, IFN-γ, TNF-α, and IL-1β levels, IgM and albumin levels	Mei et al. (2007)
Ang-1	BM	C57BL/6 mice, LPS-induced pulmonary ARDS	i.v.	2 h after injury	↓ BALF neutrophil and total cell counts, TNF-α levels, lung injury and edema, MPO activity	Xu et al. (2008)
VEGF	BM	C57BL/6 mice, elastase-induced emphysema	i.v.	14 days after injury	↑ lung function, Nrf2, HO-1, SOD mRNA levels	Chen et al. (2015b)
					↓ lung hyperinflation	
FGF-2	BM	C57BL/6 mice, LPS-induced extrapulmonary ARDS	i.v.	5 × 10^6^ cells, 1 h after injury	↓ lung injury, edema and neutrophil count, BALF proteinaceous exudate, IL-6 and TNF-α levels, MPO activity	Zhao et al. (2015)
KGF	BM	C57BL/6 mice, LPS-induced pulmonary ARDS	i.v.	5 × 10^5^ cells, 2 h after injury	↑ MSC retention in lung tissue, animal survival rate	Chen et al. (2013)
					↓ lung edema, BALF neutrophil counts, IL-1β and TNF-α levels, MPO activity, severity score	
HGF	BM	C57BL/6 mice, radiation-induced lung injury	i.v.	1 × 10^6^ cells, 6 h after injury	↓ lung injury and fibrosis, epithelial cell apoptosis, albumin and IgM levels, serum TNF-α and ICAM-1 levels, TGF-β, col1α1, and col3α1 mRNA levels	Wang et al. (2013b)
HGF	UC	BALB/c and C57BL/6 mice, bronchiolitis obliterans	i.v.	1 × 10^6^ cells, 4 h after injury	↑ IL-10 levels	Cao et al. (2016)
					↓ tracheal occlusion, cell apoptosis, IFN-γ and TGF-β levels, Treg and Th17 cell percentage	

ACE2, angiotensin-converting enzyme 2; AD, adipose tissue; Ang, angiopoietin; ARDS, acute respiratory distress syndrome; BALF, bronchoalveolar lavage fluid; BM, bone marrow; Del-1, developmental endothelial locus-1; EP2, E-prostanoid-2; FGF, fibroblast growth factor; HGF, hepatocyte growth factor; HO, heme oxygenase; IFN, interferon; IL, interleukin; iNOS, inducible nitric oxide synthase; i.t., intratracheal; i.v., intravenous; KGF, keratinocyte growth factor; LPS, lipopolysaccharide; MPO, myeloperoxidase; MSC, mesenchymal stromal cell; Nrf2; nuclear factor-erythroid 2-related factor 2; ROR2, receptor tyrosine kinase-like orphan receptor 2; SOD, superoxide dismutase; TGF, transforming growth factor; TLR, Toll-like receptor; TNF, tumor necrosis factor; UC, umbilical cord; VEGF, vascular endothelial growth factor.

Although CXCR4 and SDF-1 play a fundamental role in driving migration of MSCs to injured sites, the surface expression of the CXCR4 can decline due to successive replications, which can limit MSC homing. Overexpression of CXCR4 in BM-MSCs using a lentiviral vector was shown to enhance MSC migration *in vitro* (Yang et al., 2015). In experimental LPS-induced ARDS, administration of BM-MSCs overexpressing CXCR4 reduced lung injury, inflammation and edema, and TNF-α levels and increased IL-10 levels. BM-MSC homing in injured lung tissue was also facilitated by overexpressing CXCR4 (Yang et al., 2015). Alternatively, PGE_2_ can facilitate MSC migration by activating E-prostanoid 2 (EP2) receptor, which in turn stimulates focal adhesion kinase (FAK) and the ERK1/2 signaling pathway. When FAK and ERK1/2 were inhibited, PGE_2_-mediated BM-MSC migration was also mitigated (Lu et al., 2017). Overexpression of EP2 enhanced retention of BM-MSCs in lungs and increased IL-10 levels, but reduced IL-1β and TNF-α levels, lung injury, edema and endothelial permeability in experimental LPS-induced ARDS (Han et al., 2016b). Overexpression of the receptor tyrosine kinase-like orphan receptor 2 (ROR2) was also able to increase retention of BM-MSCs in lung tissue after LPS challenge, leading to further reduction in lung injury, edema and fibrosis, and IL-1β and IL-6 levels, compared with unmodified MSCs, in experimental LPS-induced ARDS (Cai et al., 2016).

Because MSCs can promote protection of lung tissue against oxidative injury, overexpression of antioxidant mediators, such as HO-1 and SOD, have been assessed to further enhance the therapeutic properties of MSCs. BM-MSCs expressing human HO-1 under the control of surfactant protein C promoter were harvested from transgenic mice (Liang et al., 2011). Administration of these MSCs in an experimental mouse model of hypoxia-induced pulmonary arterial hypertension led to a reduction of right ventricular hypertrophy and systolic pressure (Liang et al., 2011). Such effects were associated with inhibition of smooth muscle cell proliferation and decrease levels of inflammation-related mediators (Liang et al., 2011). When LPS-injured pulmonary endothelial cells were co-cultured with BM-MSCs overexpressing HO-1, they showed increased Nrf2 activation and attenuated NF-κB activation, leading to more effective recovery of SOD and glutathione peroxidase activity, but suppressed production of lipid peroxide, malondialdehyde (MDA), and levels of inflammation-related mediators (IL-1β, IL-6, and TNF-α) (Chen et al., 2018). In this co-culture system, levels of HGF and IL-10 produced by BM-MSCs overexpressing HO-1 were also higher after pulmonary endothelial cells were exposed to LPS (Chen et al., 2018). Compared with unmodified MSCs, administration of BM-MSCs overexpressing HO-1 further reduced lung injury, edema, neutrophilia, and levels of IL-1β and TNF-α, but increased levels of HGF, KGF, and IL-10 in serum and lung tissue, and improved the 7-day survival rate in experimental LPS-induced ARDS (Chen et al., 2019). Nrf2-overexpressing MSCs derived from amniotic membrane were also more effective than unmodified MSCs at reducing lung epithelial cell apoptosis, edema, injury, and fibrosis in experimental LPS-induced ARDS (Zhang et al., 2018b). In another study, SOD-overexpressing BM-MSCs attenuated lung epithelial cell apoptosis, inflammatory and fibrosis, and increased IL-10 levels and 30-day survival rate in radiation-induced lung injury (Chen et al., 2017a).

Several anti-inflammatory mediators targeted for genetic manipulation are produced endogenously by MSCs or stimulated by MSCs to be produced by other cells during resolution of inflammation. For instance, IL-10 upregulation has been documented extensively after MSC administration in experimental models. Interestingly, serum IL-10 levels were sustainably greater in mice infused with BM-MSCs overexpressing IL-10 compared with direct injection of IL-10 (Wang et al., 2018a). In experimental LPS-induced ARDS, BM-MSCs overexpressing IL-10 increased the presence of IL-10-producing T cells and B cells in both spleen and lung, which may protect mice against the deleterious effects of LPS challenge, thus improving the survival rate (Wang et al., 2018a). Both unmodified MSCs and UC-MSCs overexpressing IL-10 were able to improve lung static compliance in experimental *E. coli*-induced ARDS. However, UC-MSCs overexpressing IL-10 were more effective than unmodified MSCs at reducing the alveolar-arterial gradient and neutrophil infiltration in lungs, and increased the percentage of lung macrophages and their phagocytic activity (Jerkic et al., 2019). In another study, mice were subjected to lung injury by instillation of HCl, and administration of unmodified MSCs further aggravated the deleterious effects (Islam et al., 2019). Administration of MSCs overexpressing IL-10 otherwise reduced structural lung injury, fibrosis, and inflammation in experimental HCl-induced lung injury (Islam et al., 2019).

IL-1 receptor-like 1 (IL-1RL1), developmental endothelial locus-1 (Del-1), and angiotensin-converting enzyme (ACE)2 are other anti-inflammatory mediators genetically manipulated in MSCs. Overexpression of IL-1RL1, an antagonist receptor for IL-33, enhanced the therapeutic properties of AD-MSCs, leading to decreased structural lung abnormalities, IFN-γ, IL-1β, IL-33, and TLR4 mRNA levels, and increased IL-10 levels after MSCs were infused in experimental LPS-induced ARDS (Martínez-González et al., 2013). BM-MSCs overexpressing Del-1, an anti-inflammatory mediator that inhibits endothelial adhesion of leukocytes, also promoted further therapeutic benefits in experimental LPS-induced ARDS (Zhao et al., 2014b). Compared with unmodified MSCs, BM-MSCs overexpressing Del-1 were more effective at mitigating lung structural abnormalities, edema, neutrophil counts, TNF-α levels, and MPO activity (Zhao et al., 2014b). Although human MSCs do not express endogenous ACE2, a mediator that protects lung tissue against injury by counteracting the effects of angiotensin II, its introduction into UC-MSCs by lentiviral vectors further mitigated symptoms in experimental bleomycin-induced lung fibrosis (Min et al., 2015). UC-MSCs overexpressing ACE2 led to recovery of SOD and glutathione levels, reduction in MDA levels as well as lung injury and fibrosis (Min et al., 2015). In experimental LPS-induced ARDS, BM-MSCs overexpressing ACE2 were also more effective than unmodified MSCs, resulting in a greater reduction in lung injury, vascular permeability, BALF neutrophil counts, IL-1β and IL-6 levels, but increased levels of lung IL-10 and eNOS (He et al., 2015).

Other strategies that have been investigated include the overexpression of mediators that were mechanistically identified as key factors in epithelial and endothelial repair. MSC overexpression of angiopoietin (Ang)-1 or VEGF, two pro-angiogenic factors, improved disease symptoms in animal models (Mei et al., 2007; Xu et al., 2008; Chen et al., 2015b). Compared with unmodified MSCs, BM-MSCs overexpressing Ang-1 reduced BALF neutrophil and total cell counts, levels of inflammation-related mediators (IFN-γ, IL-1β, IL-6, and TNF-α), MPO activity, and alveolar proteinaceous exudate in experimental LPS-induced ARDS (Mei et al., 2007; Xu et al., 2008). In experimental elastase-induced emphysema, BM-MSCs with *cis-*resveratrol-induced Hsp70 promoter-regulated VEGFA expression were infused and demonstrated to enhance lung function and levels of VEGF and anti-oxidant mediators (HO-1, Nrf2 and SOD), and reduced lung structural abnormalities (Chen et al., 2015b). A subset of placenta-derived MSCs expressing platelet-derived growth factor (PDGF) receptor-β also demonstrated greater expression of pro-angiogenic factors (Ang-1, Ang-2, FGF, PDGF, and VEGF) as well as an increased proliferation rate and wound repair ability in comparison with MSCs not expressing PDGR receptor-β (Wang et al., 2018b). Furthermore, dental pulp-derived MSCs overexpressing HIF-1α demonstrated enhanced angiogenic ability by increasing the amount of the Notch ligand Jagged1 loaded into their EVs (Gonzalez-King et al., 2017).

Compared with unmodified MSCs, administration of BM-MSCs overexpressing FGF-2 was more effective at reducing lung injury, edema, neutrophil counts, MPO activity, and TNF-α levels in experimental LPS-induced ARDS (Zhao et al., 2015). Overexpression of KGF also enhanced the therapeutic actions of BM-MSCs in experimental LPS-induced ARDS, leading not only to improved pulmonary vascular permeability but also mitigated pro-inflammatory responses, which resulted in a reduction in the severity score and mortality rate (Chen et al., 2013). Furthermore, the therapeutic effects of MSCs were enhanced by overexpressing HGF. When co-cultured with MSCs, dendritic cells were converted into the regulatory profile in a mechanism dependent on MSC-secreted HGF (Lu et al., 2019). MSCs overexpressing HGF induced immune tolerance, thus reducing dendritic cell aggregation in the lung tissue of experimental LPS-induced ARDS (Lu et al., 2019). In experimental radiation-induced lung injury, BM-MSCs overexpressing HGF decreased lung histopathologic abnormalities, epithelial cell apoptosis, and mRNA levels of inflammatory (ICAM, IFN-γ, IL-6, and TNF-α) and fibrotic factors (col1α1, col3α1, and TGF-β) (Wang et al., 2013b). Overexpression of HGF in UC-MSCs reduced tracheal occlusion and apoptosis, IFN-γ and TGF-β mRNA levels in allograft trachea, and the percentage of Treg cells and the ratio T_H_1/T_H_2 in the spleen of experimental bronchiolitis obliterans (Cao et al., 2016). HGF-overexpressing MSCs were also able to protect lung tissue against injury by HCl instillation (Islam et al., 2019).

Engineered MSCs that overexpress any of the aforementioned factors have been shown to efficiently potentiate therapeutic actions by simultaneously increasing the expression levels of the cited protein and secreting other paracrine immunomodulatory mediators in lung tissue. Furthermore, genetically engineered cells were used in patients with pulmonary arterial hypertension and some positive effects were observed in an early-stage clinical trial (Granton et al., 2015). Nevertheless, genetic modification is usually performed with a viral vector to achieve high transduction efficacy. For such an approach, it can take a few months to acquire genetically modified MSCs for harvesting in sufficient number for infusion, which might limit their use for acute respiratory diseases.

## 3 Insights from early-stage clinical investigations of MSC-based therapy

MSCs are able to avoid host immune surveillance (Le Blanc et al., 2003; Ankrum et al., 2014; De Witte et al., 2016) and have a long-established safety record. To the best of our knowledge, assessments of MSC preconditioning strategies are still under investigation in pre-clinical studies and there is no clinical trial using preconditioned MSCs in patients with respiratory diseases. Several clinical trials are currently investigating non-preconditioned MSC-based therapy for respiratory diseases, in particular for COVID-19 (da Silva et al., 2021). However, despite some positive effects, clinical benefits were not clearly demonstrated in early-stage clinical studies of MSCs in ARDS and COPD (Weiss et al., 2013; Matthay et al., 2019).

Sixty-two patients with moderate to severe COPD were enrolled in a placebo-controlled, randomized phase 2 clinical trial (Weiss et al., 2013). Although systemic infusion of BM-MSCs was well tolerated and safe, no differences in lung function assessments and the 6-min walking test were observed among the cohorts. However, patients who had increased circulating C-reactive protein (CRP) levels at study entry demonstrated a reduction in CRP levels after treatment with BM-MSCs (Weiss et al., 2013). In a *post hoc* analysis of clinical trial data, patients were stratified according to the baseline CRP levels (Weiss et al., 2021). Surprisingly, patients with COPD with higher baseline CRP levels (≥4 mg/L) treated with BM-MSCs demonstrated significant improvements in forced vital capacity, forced expiratory volume in 1 s, and the 6-min walking test at 120 days (Weiss et al., 2021). Although variable, these improvements persisted over the 2-year observation period (Weiss et al., 2021).

Sixty patients with moderate to severe ARDS were enrolled in a double-blind, randomized phase 2a clinical trial (Matthay et al., 2019). Intravenous infusion of allogenic BM-MSCs did not induce hemodynamic or respiratory side effects, and thus proved to be safe. Although MSC efficacy could not be properly supported and this was attributed to reduced MSC viability after thawing, patients treated with BM-MSCs with greater viability after thawing demonstrated a reduction in plasma Ang-2 levels after 6 h (Matthay et al., 2019). A subsequent analysis of these data identified a nested cohort of patients with ARDS treated with BM-MSCs who presented a significant reduction of airspace total protein, Ang-2, IL-6, and TNF-R1 levels within 48 h after infusion of BM-MSCs (Wick et al., 2021).

Similar findings have been observed in clinical trials using MSCs to treat graft-versus-host disease (GvHD). Fifty-five patients with GvHD were enrolled in a multicenter phase 2 clinical trial. No side effects during or immediately after MSC infusion were reported. A sub-population demonstrated a complete response and had lower transplantation-associated mortality 1 year after MSC infusion compared with patients who had only partial or no response (Le Blanc et al., 2008). A retrospective study analyzed a cohort of 37 children with GvHD treated with BM-MSCs, and a significant increase in survival rate was reported in complete responders compared with partial and non-responders (Ball et al., 2013).

These findings support the hypothesis of stratifying patients according to disease sub-phenotypes (Matthay et al., 2020; Vasquez et al., 2021) to identify those who are most likely to benefit from MSC therapy. In this context, some studies have applied integrated genomic approaches or cluster analysis to define sub-phenotypes in patients with sepsis (Davenport et al., 2016; Scicluna et al., 2017) and ARDS (Bos et al., 2017). Patient stratification and treatment in a personalized fashion have also been successfully performed in other diseases, such as CF (Lopes-Pacheco, 2020; Lopes-Pacheco et al., 2021). Moreover, determination of a panel of biomarkers that more precisely differentiate disease sub-phenotypes may facilitate the identification of patients who might best respond to MSC therapy.

## 4 Translational challenges and future directions

Despite the significant progress in assessing MSC-based therapy for respiratory diseases, there is a lack of consistency among the different experimental studies, which might also play a role in the limited success in clinical investigations. For instance, increasing evidence has shown that MSCs from different sources retain certain organ-specific functions and properties, including gene expression and stability, cell-surface proteins, differentiation patterns, secreted cytokine profile, and immunomodulatory ability (Ostanin et al., 2011; Viero Nora et al., 2012; Elahi et al., 2016; Heo et al., 2016; Sacchetti et al., 2016). Accordingly, such differences may have a major impact on MSC survival and therapeutic properties (Wagner et al., 2007; Antunes et al., 2014; Chao et al., 2014; Silva et al., 2018), and further experimental studies should be carried out to comparatively assess the effects of MSCs from different sources and clarify whether cells from one source may promote greater therapeutic responses than others for respiratory diseases.

The influence of donor intrinsic variability on the properties of MSCs has been highlighted in recent studies. MSCs from different donors preconditioned or not with cytokines and hypoxia exhibited a variable degree of immunomodulatory actions (François et al., 2012a; Amati et al., 2017; Kang et al., 2018). MSCs harvested from donors of different ages also demonstrated distinct *in vitro* differentiation potential and proliferation rates (Zaim et al., 2012; Mehrian et al., 2020). The infusion of aged MSCs or their EVs led to impaired immunomodulatory actions (Yu et al., 2014; Huang et al., 2019) or even deleterious effects in disease models (Lee and Yu, 2020). Moreover, certain diseases can alter MSC niche or cell metabolism, thus promoting a negative impact on their therapeutic ability when they are harvested for either autologous or allogeneic infusion (Silva et al., 2014; Antebi et al., 2018; Kim et al., 2020; Antunes et al., 2021). To identify MSCs of potential clinical use, a number of reference biomarkers should be validated for donor selection and thus the number of batches of good donors should be maximized. Nevertheless, scalability remains limited because successive replications of MSCs and long-term culture to acquire large quantities of cells may affect their properties (Lee et al., 2009; Zaim et al., 2012). The development of scalable platforms, such as bioreactors, is urgently needed to generate a higher number of good-quality MSCs for clinical use.

Some conflicting data exist on whether the freezing/thawing process may be detrimental to the cells. Although some studies have indicated that both freshly thawed and continuously cultured MSCs have similar anti-inflammatory activities (Cruz et al., 2015; Tan et al., 2019; Horie et al., 2020b), others have suggested that freshly thawed MSCs are less effective than continuously cultured MSCs (François et al., 2012b; Chinnadurai et al., 2016). One report also argued that cryopreserved MSCs may require a recovery period before they may be infused (François et al., 2012b). Further studies should investigate the effects of continuously cultured and freshly thawed MSCs in disease-specific models. Meanwhile, MSC cryopreservation and storage conditions should be optimized to prevent reduction in their viability (Matthay et al., 2019). This is of particular relevance for acute respiratory diseases, which occur with rapid onset and progression and there is insufficient time for expanding MSCs. Overall, there is a lack of standardization in MSC manufacturing practices among different facilities and academic centers in the United States and Europe (Trento et al., 2018; Phinney et al., 2019). Accordingly, well-standardized and regulated manufacturing practices need to be successfully implemented among MSC distributors (Fernández-Santos et al., 2022). Specific legislation should be also established for the commercialization if cell therapy products are to become a ready-to-use therapy.

Although most clinical investigations have been using systemic infusions of MSCs, there is no consensus on the optimal delivery route. Differences in the early biodistribution of MSCs were observed after their infusion *via* different routes (Cardenes et al., 2019). Systemic infusion (e.g., intravenous) ensures a broad distribution of cells throughout the body, and local infusion (e.g., intratracheal or endobronchial) can deliver MSCs directly into the pulmonary compartments. Both delivery routes might provide specific advantages for each respiratory disease, but only a few experimental studies have comparatively assessed different delivery routes and therapeutic responses were equivalent (Curley et al., 2013; Antunes et al., 2014; Alcayaga-Miranda et al., 2015; Devaney et al., 2015). The therapeutic window and index of MSC infusion have been also investigated. In experimental models, further therapeutic responses were observed when MSCs were infused soon after the initial injury (Chang et al., 2012; Hayes et al., 2015; Horie et al., 2020b), which suggests that MSCs might be more effective in acute inflammatory conditions. On the other hand, MSCs were less effective in promoting repair when tissue remodeling was already established (Lopes-Pacheco et al., 2014; Mariñas-Pardo et al., 2014; Sabry et al., 2014; Kitoko et al., 2018). One potential explanation for this limited tissue repair is the short period of permanence of cells in the injured tissue, which might be overcome, at least partially, by preconditioning methods that enhance MSC retention (as described in previous sections) or repeated infusions of MSCs. In this context, multiple infusions of cells were demonstrated to maintain therapeutic benefits for a longer period and promoted a more efficient tissue repair in various experimental models (Lopes-Pacheco et al., 2013; Poggio et al., 2018; Horie et al., 2020b; Castro et al., 2020).

Another important fact to be considered is the co-application of other therapies with MSCs. Many respiratory diseases can be caused by or predispose to infections, therefore MSCs have been used concomitantly with antibiotics in experimental models, and therapeutic responses were superior with combined therapy (Mei et al., 2010; Alcayaga-Miranda et al., 2015; Sung et al., 2016; Horie et al., 2020b). Synergistic effects were also observed with the combination of MSCs with *N-*acetylcysteine (Shin et al., 2019) or pre-activated disaggregated platelets (Chen et al., 2017b). On the other hand, extracorporeal membrane oxygenation (ECMO) is a therapeutic modality frequently used in critical care illness. In an *ex vivo* model, increased pressure and reduced flow through the apparatus were observed after MSC infusion because cells were adhering to membrane oxygenation fibers (Millar et al., 2019; von Bahr et al., 2019). Further studies should be performed to determine potential synergistic and interference effects of MSCs with other therapies commonly used in patients with respiratory diseases.

## 5 Outlook and conclusion

MSCs have been investigated extensively over the last decade as a potential therapy for numerous diseases. However, despite the promising therapeutic effects observed in experimental models, MSC efficacy has fallen far short in most clinical trials for respiratory diseases. Several functional enhancement strategies have been investigated to potentiate the therapeutic actions of MSCs and overcome the limitations associated with reduced MSC efficacy in clinical trials. Genetic manipulation and preconditioning methods can target different properties of MSCs, such as homing, survival, and immunomodulatory activities, and they may be used individually or in combination to further enhance therapeutic outcomes. Patients may not respond equally to MSC therapy, and their stratification based on disease sub-phenotypes may facilitate identification of those who might best benefit from MSC therapy. The environmental conditions of disease can have a major impact on the therapeutic actions of MSCs, therefore different MSC preconditioning strategies can be used to treat disease sub-phenotypes in a personalized medicine approach. Although MSCs still hold immense promise due to their multifaceted mechanisms of action and therapeutic abilities, it has become evident that further experimental research is required to better understand the optimal way to use MSCs and how to translate this information into clinical trials to achieve the greatest clinical outcomes.
